# Identification of a Costimulatory Molecule-Related Signature for Predicting Prognostic Risk in Prostate Cancer

**DOI:** 10.3389/fgene.2021.666300

**Published:** 2021-08-16

**Authors:** Shengdong Ge, Xiaoliang Hua, Juan Chen, Haibing Xiao, Li Zhang, Jun Zhou, Chaozhao Liang, Sheng Tai

**Affiliations:** ^1^Department of Urology, The First Affiliated Hospital of Anhui Medical University, Hefei, China; ^2^Anhui Province Key Laboratory of Genitourinary Diseases, Anhui Medical University, Hefei, China; ^3^The Institute of Urology, Anhui Medical University, Hefei, China; ^4^The Ministry of Education Key Laboratory of Clinical Diagnostics, School of Laboratory Medicine, Chongqing Medical University, Chongqing, China

**Keywords:** prostate cancer, costimulatory molecules, bioinformatics, biomarker, prognostic signature

## Abstract

Costimulatory molecules have been proven to enhance antitumor immune responses, but their roles in prostate cancer (PCa) remain unexplored. In this study, we aimed to explore the gene expression profiles of costimulatory molecule genes in PCa and construct a prognostic signature to improve treatment decision making and clinical outcomes. Five prognosis-related costimulatory molecule genes (RELT, TNFRSF25, EDA2R, TNFSF18, and TNFSF10) were identified, and a prognostic signature was constructed based on these five genes. This signature was an independent prognostic factor according to multivariate Cox regression analysis; it could stratify PCa patients into two subgroups with different prognoses and was highly associated with clinical features. The prognostic significance of the signature was well validated in four different independent external datasets. Moreover, patients identified as high risk based on our prognostic signature exhibited a high mutation frequency, a high level of immune cell infiltration and an immunosuppressive microenvironment. Therefore, our signature could provide clinicians with prognosis predictions and help guide treatment for PCa patients.

## Introduction

Over the past few years, prostate cancer (PCa) has become one of the most common lethal malignant tumors in men, posing a grave danger to human health (Nguyen-Nielsen and Borre, 2016). The latest global cancer statistics predicted approximately 191,930 new cases of PCa and 33,330 PCa-related deaths in America in 2020 (Siegel et al., 2020). Early stage PCa has a good prognosis after surgical resection, but once it recurs or develops into metastatic castration-resistant prostate cancer (mCRPC), there are fewer treatment options available, and the average survival time is only 2–3 years (Omlin et al., 2013; Wang et al., 2018). With advancements in medicine, various targeted therapies and immunotherapies have further improved the prognosis of PCa, especially metastatic PCa. However, only a small percentage of PCa patients can benefit from these therapies (Sharma et al., 2017). Epigenetic regulation of gene plays a critical role in cancer evolution (Grasso et al., 2012), and gene expression variations or mutations have been frequently observed in PCa (Gu et al., 2015). Therefore, exploring new genetic and epigenetic biomarkers will be useful for predicting specific survival of PCa patients and improving clinical outcomes.

Immunotherapy has become an important cancer treatment method that can activate the immune system to attack and kill tumor cells. In particular, immune checkpoint inhibitor (ICI) therapy has seen great success in multiple types of cancer (Loos et al., 2008; Jansen et al., 2019). However, only a minority of PCa patients have an obvious response to immunotherapy, stressing the need to explore more effective treatment methods (Sharma et al., 2017). Studies have shown that the tumor microenvironment (TME), which includes immune cells, stromal cells, mesenchymal cells, cytokines, chemokines, and blood vessels, plays an important role in the process of tumorigenesis and development (Casey et al., 2015). Thus, a deeper understanding of the immune microenvironment will help us improve PCa patient outcomes. In fact, many studies are currently exploring the therapeutic potential of costimulatory molecules in cancer (Wei et al., 2018). Costimulatory molecules play an important role in the regulation of tumor immunity by affecting the activation, proliferation, survival and secretion of T cells (Croft et al., 2013; Schildberg et al., 2016). At present, there are two main families of costimulatory molecules: the B7-CD28 family and the tumor necrosis factor (TNF) family. The most common ICIs target programmed cell death protein 1 (PD-1) and programmed cell death 1 ligand 1 (PD-L1), which are CD28 and B7 family members, respectively (Zhang et al., 2018). The latest research showed that TNF family members, including 19 TNF ligand superfamily (TNFSF) members and 29 TNF receptor superfamily (TNFRSF) members, are also potential therapeutic targets that play important roles in the regulation of cellular functions. TNFSF/TNFRSF members can control immune cells to coordinate various mechanisms that drive the costimulation or cosuppression of immune responses (Dostert et al., 2019). However, the molecular functions of these costimulatory molecules in PCa remain unexplored. Therefore, we aimed to develop a specific and effective prognostic signature based on several costimulatory molecules to guide treatment and improve the clinical outcomes of PCa.

High-throughput sequencing and bioinformatics technologies have facilitated the identification of key pivotal biomarkers for PCa (Wang et al., 2013; Gu et al., 2020). Here, we acquired 60 costimulatory molecules (comprising 12 B7-CD28 family members and 48 TNF family members) as candidates for potential molecular therapy targets (Janakiram et al., 2015; Dostert et al., 2019). Subsequently, the mRNA sequencing data and clinical information of PCa patients were downloaded from The Cancer Genome Atlas (TCGA) database to predict disease free survival, and a prognostic signature for PCa patients was constructed. We validated the efficiency of the prognostic signature in four external datasets. Previous studies indicated that the composition of infiltrating immune cells in the tumor microenvironment is fundamental to the outcomes of immunotherapy (Wu et al., 2020). Thus, understanding immune infiltration in the PCa microenvironment is an essential prerequisite for the success of immunotherapy. In our study, we systematically described the landscape of costimulatory molecules and classified PCa patients into different groups according to the prognostic signature. We further evaluated the immune microenvironment of PCa patients to identify patients who may benefit more from immunotherapy.

## Materials and Methods

### Data Acquisition and Preprocessing

**FIGURE 1:**
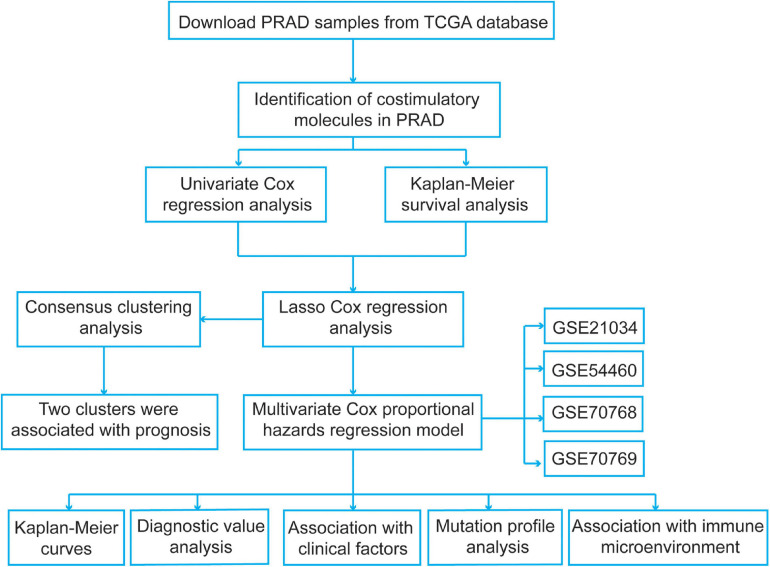
The flowchart of the present study design.

We identified several pivotal prognosis-related costimulatory molecule genes in PCa using comprehensive bioinformatics analysis. The specific flow chart is shown in Figure 1. The mRNA expression profiles of PCa patients in the TCGA database were obtained from UCSC Xena^1^. A total of 52 normal samples and 498 PCa samples were obtained. Disease-free survival information was obtained from the cBioPortal for Cancer Genomics^2^. All expression data were normalized using the “RNA-Seq by Expectation-Maximization” package, and log_2_(x + 1) transformation was performed. To select genes with prognostic values and establish a risk signature, samples from PCa patients with unknown survival status or without follow-up information were excluded, and a total of 491 PCa samples were included. Then, we obtained four normalized independent microarray datasets, namely, GSE21034, GSE54460, GSE70768, and GSE70769, from the Gene Expression Omnibus (GEO^3^) database. The GSE21034, GSE54460, GSE70768, and GSE70769 datasets included 140 PCa samples, 90 PCa samples, 111 PCa samples, and 92 PCa samples, respectively. For the GSE21034 dataset, the expression data were normalized according to the robust multi-array average (RMA) method, and the probes were annotated using the corresponding annotation file. For the GSE54460 dataset, the expression data were expressed as fragments per kilobase per million values. The GSE70768 and GSE70769 datasets were sequenced using the Illumina HumanHT-12 V4.0 expression BeadChip platform, and the probes were annotated using the corresponding “illuminaHumanv4.db” R package. The expression data in these two datasets were log2 transformed and quantile normalized. The detailed information for the datasets used in present study were showed in Supplementary Table 1.

### Identification of Costimulatory Molecules With Prognostic Significance in PCa

These costimulatory molecule genes were mapped to the TCGA dataset, and univariate Cox regression analysis was performed to identify costimulatory molecule genes with prognostic significance in PCa with *P* < 0.05. We also used Kaplan–Meier curves and log-rank tests to verify the prognostic values of these survival-related costimulatory molecule genes using the “survival” R package (Tibshirani, 1997). Least absolute shrinkage and selection operator (LASSO) Cox regression analysis was used to screen the most significant prognostic costimulatory molecule genes in PCa using the “glmnet” R package. The optimal values of penalty parameters were determined by 10-fold cross-validation.

### Consensus Clustering of Prognosis-Related Costimulatory Molecule Genes

To further explore the functions and prognostic values of the costimulatory molecule genes in PCa from the results of LASSO Cox regression analysis, we carried out consensus clustering to confirm the cluster numbers using the “Consensus ClusterPlus” R package. Then, Kaplan-Meier curves were plotted to verify the prognostic values of the clusters. In addition, gene set enrichment analysis (GSEA) was also performed to reveal the potential functional mechanisms using the h.all.v7.2.symbols.gmt file. False discovery rate (FDR) < 0.25 and normalized *P*-value < 0.05 were set as the threshold values for significance.

### Construction of a Prognostic Signature Based on the Five Survival-Related Costimulatory Molecule Genes

We performed multivariate Cox proportional hazards regression analysis of these five survival-related costimulatory molecule genes to obtain the corresponding coefficients. Subsequently, a prognostic signature was constructed, and the risk score was calculated using the following formula:

$$\text{Risk score}=\beta_{1}*{\text{Exp}}_{1}+\beta_{2}*{\text{Exp}}_{2}+\beta_{i}*{\text{Exp}}_{i}.$$

β and *Exp* represent the coefficients from the multivariate Cox proportional hazards regression analysis and the expression levels of selected genes, respectively. In addition, we generated Kaplan–Meier curves and receiver operating characteristic (ROC) curves to evaluate and validate the efficiency of the signature. Then, we performed principal component analysis (PCA) to estimate the distribution patterns and confirm the cluster numbers in the TCGA dataset by using the “ggplot2” R package.

### Functional Enrichment Analysis

To explore signature-related biological pathways, genes that were strongly correlated with the risk score (correlation coefficient *R* > 0.4) were obtained. A total of 525 positively correlated genes and 87 negatively correlated genes were generated. We performed gene ontology (GO) and Kyoto Encyclopedia of Genes and Genomes (KEGG) enrichment analyses to investigate the possible molecular mechanisms of the risk signature genes using the Database for Annotation, Visualization and Integrated Discovery version 6.8^4^. *P* < 0.05 was regarded as the cutoff value.

### Estimation of the Immune Microenvironment Composition

For quantification of the cellular composition of the immune infiltrates in each risk group, a set of metagenes, including non-overlapping sets of genes that are representative of twenty-eight specific immune cell subpopulations, was obtained from a previous study (Charoentong et al., 2017). Then, we employed single-sample gene set enrichment analysis (ssGSEA) to quantify the 28 types of immune cells based on the metagenes. In the tumor microenvironment, immune and stromal cells are the two main non-tumor components and have been proposed to be valuable for tumor treatment and prognostication. To assess the tumor microenvironment of different risk groups, the immune and stromal scores for the TCGA dataset, reflecting the infiltration levels of non-tumor cells, were calculated using the ESTIMATE package (Yoshihara et al., 2013). The differences in immune cell composition and immune and stromal scores were compared between high-risk and low-risk PCa patients.

### Comparison of Significantly Mutated Genes

Tumor mutation burden (TMB) is defined as the total amount of coding errors of somatic genes, base substitutions, insertions or deletions detected per million bases (Chalmers et al., 2017). In the present study, we used 38 MB as the length of exons. TMB was calculated as the number of variants/the length of the exons for each PCa sample via Perl scripts based on the JAVA8 platform (Abida et al., 2019). The somatic mutation status data of PCa samples (workflow type: VarScan2 Variant Aggregation and Masking) were downloaded from the TCGA data portal^5^ in December 2020. Mutation data were filtered using the “maftools” R package and compared between high-risk and low-risk patients.

### Statistical Analyses

We performed the *t*-test or Wilcoxon test for comparisons between the two groups, and used the one-way analysis of variance test or Kruskal–Wallis test for comparison between multiple samples. Univariate and multivariate Cox regression analyses were performed to evaluate the prognostic values of costimulatory molecule genes. Moreover, we used Kaplan–Meier curves and log-rank tests to assess survival differences. Pearson’s chi-square test was used to assess differences in the distribution of clinical variables for PCa patients. All procedures involved in the present study were conducted using Perl and R scripts. A *P*-value < 0.05 was considered to indicate significance in all statistical tests.

## Results

### Identification of Costimulatory Molecule Genes With Prognostic Significance in PCa

**FIGURE 2:**
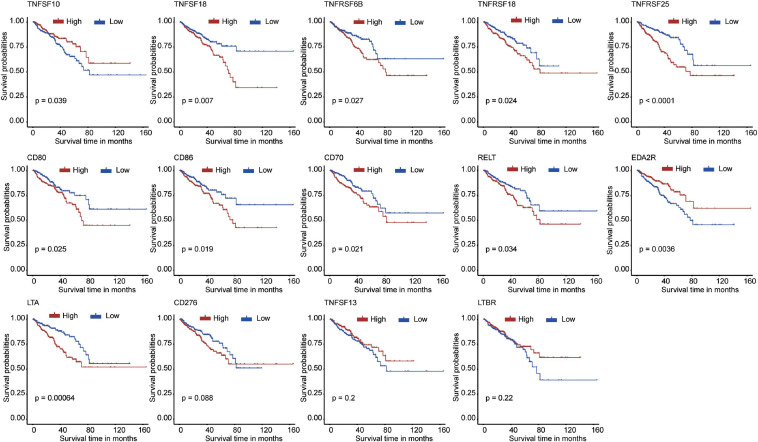
The Kaplan–Meier curves for the 14 costimulatory molecule genes in prostate cancer from The Cancer Genome Atlas dataset, including TNFSF10, TNFSF18, TNFRSF6B, TNFRSF18, TNFRSF25, CD80, CD86, CD70, RELT, EDA2R, LTA, CD276, TNFSF13, and LTBR.

First, we extracted the expression data of 60 costimulatory molecule genes in PCa from the TCGA database. These costimulatory molecules consisted of 13 B7-CD28 family genes and 47 TNF family genes, and the gene expression correlations among the 60 costimulatory molecule genes are shown in Supplementary Figure 1. Then, we performed a univariate Cox regression analysis to assess the prognostic relevance of the expression of these costimulatory molecule genes, and the screening criterion was *P* < 0.05. The results showed that a total of 14 costimulatory molecule genes were significantly associated with the prognosis of PCa. Among these fourteen significant genes, ten genes (TNFSF18, TNFRSF6B, TNFRSF18, TNFRSF25, CD80, CD86, CD70, RELT, LTA, and CD276) were recognized as risk factors with HR > 1, and four genes (TNFSF10, EDA2R, TNFSF13, and LTBR) were recognized as protective factors with HR < 1 (Table 1). Moreover, Kaplan-Meier curves were used to confirm the prognostic value of each of the fourteen genes (Figure 2). The results showed that high expression of the risk genes (TNFRSF18, TNFRSF6B, TNFSF18, TNFRSF25, CD80, CD86, CD70, RELT, and LTA) was associated with a poor prognosis in PCa. High expression of protective genes (TNFSF10 and EDA2R) was associated with a good prognosis in PCa. However, patients with high expression of CD276, TNFSF13, and LTBR had no significant difference in prognosis compared with patients with low expression. In addition, LASSO Cox regression analysis was executed to select genes with the highest prognostic value; five genes were selected, namely, RELT, EDA2R, TNFSF10, TNFSF18, and TNFRSF25 (Supplementary Figures 2A,B).

**TABLE 1:** Univariate Cox analysis of costimulatory molecule genes in TCGA dataset.

Official symbol	HR	*P*-value	HR.95L	HR.95H
RELT	1.649	<0.001	1.319	2.061
TNFRSF25	1.418	<0.001	1.196	1.682
EDA2R	0.746	<0.001	0.635	0.877
TNFSF18	1.703	<0.001	1.269	2.284
TNFRSF18	1.321	0.001	1.129	1.546
CD80	1.317	0.002	1.107	1.567
CD276	1.652	0.002	1.196	2.284
LTA	1.277	0.005	1.080	1.510
TNFRSF6B	1.346	0.005	1.095	1.656
CD86	1.336	0.006	1.087	1.641
TNFSF10	0.742	0.015	0.584	0.943
LTBR	0.572	0.018	0.360	0.908
CD70	1.243	0.027	1.025	1.507
TNFSF13	0.699	0.037	0.499	0.979
LTB	1.135	0.051	0.100	1.289
TNFRSF4	1.167	0.055	0.997	1.367
TNFRSF8	1.207	0.072	0.983	1.482
TNFRSF1B	1.238	0.080	0.974	1.574
TNFRSF10C	0.859	0.080	0.724	1.019
EDA	1.186	0.086	0.976	1.440
CTLA4	1.136	0.089	0.981	1.316
TNFSF13B	1.163	0.090	0.977	1.385
FASLG	1.136	0.144	0.957	1.348
TNFSF15	0.911	0.150	0.803	1.034
TNFRSF10D	0.877	0.161	0.731	1.053
ICOS	1.108	0.164	0.959	1.280
TNFSF14	1.136	0.179	0.943	1.369
TNFRSF9	1.122	0.183	0.947	1.328
TNFRSF10A	0.873	0.188	0.714	1.069
TNFSF11	1.150	0.200	0.928	1.426
TNFRSF1A	0.755	0.213	0.485	1.175
TNFSF9	1.098	0.227	0.944	1.277
TNFRSF12A	1.089	0.316	0.922	1.287
TNFRSF13B	1.069	0.318	0.937	1.220
TNFRSF17	1.071	0.323	0.935	1.227
CD27	1.076	0.352	0.922	1.255
NGFR	0.935	0.370	0.807	1.083
PDCD1	1.074	0.397	0.910	1.268
FAS	0.903	0.405	0.710	1.149
TNFSF8	1.070	0.422	0.907	1.263
TNFSF12	0.872	0.435	0.619	1.229
TNFSF4	1.115	0.445	0.843	1.476
TNF	1.064	0.470	0.899	1.258
HHLA2	0.944	0.500	0.799	1.115
TNFRSF11A	0.939	0.516	0.778	1.134
ICOSLG	1.114	0.552	0.800	1.592
VTCN1	0.971	0.558	0.881	1.071
TNFRSF13C	1.056	0.565	0.877	1.271
TNFRSF11B	0.951	0.572	0.800	1.132
TNFRSF10B	0.897	0.585	0.607	1.326
CD274	1.052	0.601	0.869	1.274
TNFRSF21	1.066	0.612	0.833	1.364
TNFRSF14	1.078	0.627	0.797	1.457
TMIGD2	1.044	0.633	0.876	1.244
EDAR	1.030	0.634	0.911	1.165
PDCD1LG2	1.023	0.817	0.842	1.245
CD28	0.984	0.840	0.844	1.148
TNFRSF19	1.009	0.899	0.882	1.153
CD40	0.988	0.907	0.811	1.204
CD40LG	0.995	0.948	0.851	1.163

*HR, hazard ratio; HR.95L, Lower limit of the 95% confidence interval; HR.95H, Upper limit of the 95% confidence interval.*

### Identification of Two Clusters Using Consensus Clustering

**FIGURE 3:**
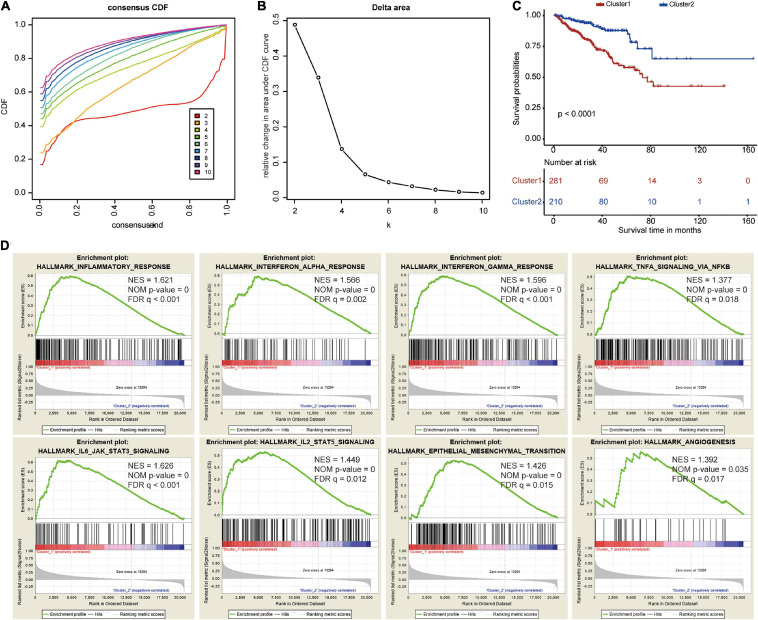
Consensus clustering based on the five costimulatory molecule genes. **(A)** Consensus clustering cumulative distribution function (CDF) for *k* = 2 to *k* = 10. **(B)** The relative change in area under the CDF curve for *k* = 2 to *k* = 10. **(C)** The Kaplan–Meier curve evaluate the prognosis of prostate cancer patients. **(D)** The Gene Set Enrichment Analysis showed that several oncogenic pathways were significantly enriched in cluster 1.

To explore the prognostic value of the five costimulatory molecule genes, we performed a consensus clustering analysis to stratify PCa patients. Then, we executed a PCA to validate the reliability of the cluster numbers. The results demonstrated that *k* = 2 was the most stable value in the TCGA dataset (Figures 3A,B). The clustering heatmap and PCA clearly demarcated two clusters when *k* = 2 (Supplementary Figures 3A,B). Nevertheless, we also showed the results of consensus clustering when *k* = 3 (Supplementary Figure 3C), *k* = 4 (Supplementary Figure 3E), and *k* = 5 (Supplementary Figure 3G). The clusters overlapped with each other when there were three (Supplementary Figure S3D), four (Supplementary Figure 3F), or five (Supplementary Figure 3H) clusters. Therefore, patients were divided into two clusters (cluster 1 and cluster 2). The Kaplan–Meier curves revealed that the two clusters had different prognoses, and cluster 1 showed a worse prognosis than cluster 2 (Figure 3C). In addition, GSEA revealed that several oncogenic pathways, including the inflammatory response (normalized enrichment score: NES = 1.621), the interferon alpha response (NES = 1.566), the interferon gamma response (NES = 1.596), TNFA signaling via NFKB (NES = 1.377), IL6/JAK/STAT3 signaling (NES = 1.626), IL2/STAT5 signaling (NES = 1.449), epithelial mesenchymal transition (NES = 1.426), and angiogenesis (NES = 1.392), were significantly enriched in cluster 1 (Figure 3D).

### Validation of the Five Survival-Related Costimulatory Molecule Genes

**FIGURE 4:**
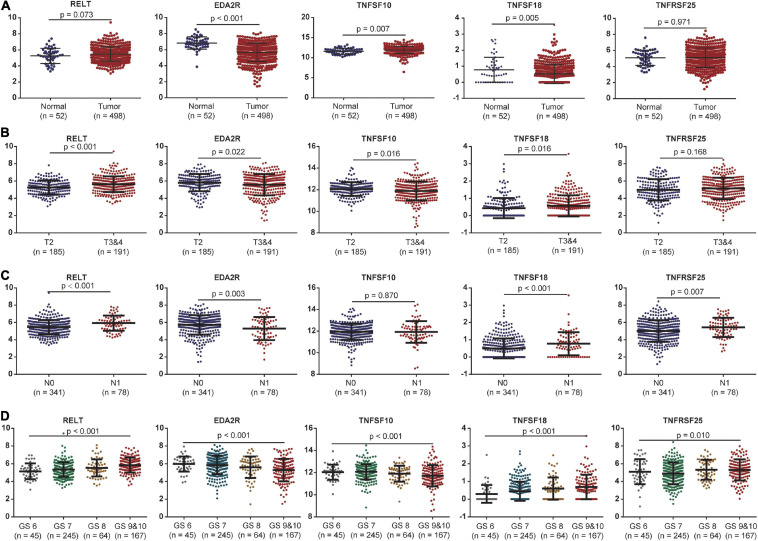
The association between the expression levels of the five costimulatory molecules and clinical factors. **(A)** The expression levels of the five costimulatory molecules between the normal and tumor samples; **(B)** T2 staging vs. T3 and T4 staging; **(C)** N0 staging vs. N1 staging. **(D)** Gleason score for 6, 7, 8, 9, and 10. GS represents the Gleason score. The unpaired Student’s *t*-test was performed for comparison between two samples and the one-way analysis of variance test for comparison between multiple samples.

The expression levels of these five costimulatory molecule genes in PCa were compared between normal and tumor samples. RELT and TNFSF10 had high expression levels in tumor tissues compared with normal tissues, while EDA2R and TNFSF18 had low expression levels in tumor tissues compared with normal tissues in TCGA dataset. TNFRSF25 expression was not significantly difference between tumor tissues and normal tissues in the TCGA dataset (Figure 4A). In addition, we assessed the correlations between the expression levels of these five genes in different clinical subgroups. RELT and TNFSF18 were expressed at high levels, and EDA2R and TNFSF10 were expression at low levels in patients with advanced disease in terms of T stage. The expression level of TNFRSF25 showed no significant difference among patients with different T stages (Figure 4B). The expression levels of RELT, TNFSF18, and TNFRSF25 were high, and the expression levels of EDA2R were low in patients with lymphatic metastasis (Figure 4C). The expression levels of RELT, TNFSF18, and TNFRSF25 were high, and the expression levels of EDA2R and TNFSF10 were low in patients with a high Gleason score (Figure 4D).

### Construction and Validation of the Prognostic Signature Based on Five Costimulatory Molecule Genes

We performed a multivariate Cox proportional hazards regression analysis based on these five survival-related costimulatory molecule genes. Subsequently, we constructed a prognostic model to stratify PCa patients based on the above multivariate Cox regression analysis results, integrating the expression profiles of five survival-related costimulatory molecule genes and their corresponding regression coefficients. A risk score was calculated as shown below:

Riskscore=(0.23799*RELT)+(-0.25874*EDA2R)

+(-0.25207*TNFSF10)+(0.45293*TNFSF18)

+(0.25466*TNFRSF25)

**FIGURE 5:**
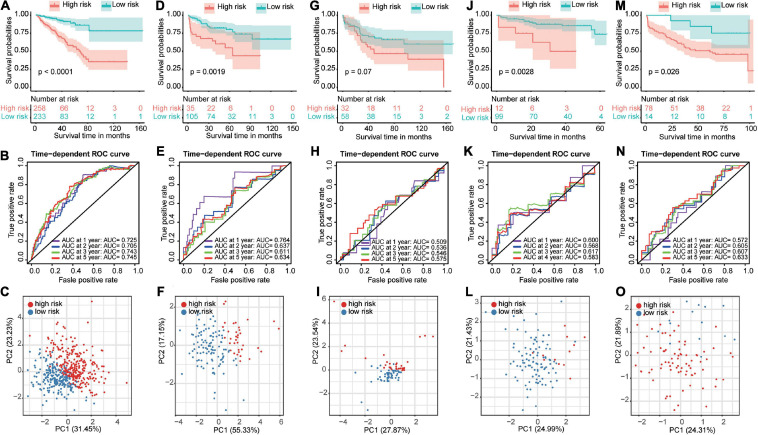
Construction and validation of a prognostic-related risk score model. The Kaplan–Meier curves, time-dependent receiver operating characteristic curves and principal component analysis for The Cancer Genome Atlas dataset **(A–C)**, GSE21034 dataset **(D–F)**, GSE54460 dataset **(G–I)**, GSE70768 dataset **(J–L)**, and GSE70769 dataset **(M–O)**.

Next, we used the optimal cutoff point for survival to stratify PCa patients into the high-risk and low-risk groups in all datasets. Kaplan–Meier analysis revealed that patients in the high-risk group had a significantly poorer prognosis than patients in the low-risk group (Figure 5A). We further validated these results in four GEO datasets. Similarly, significant differences were found in the GSE21034 (Figure 5D), GSE70768 (Figure 5J), and GSE70769 (Figure 5M) datasets. The same trend, although less significant difference, was observed in the GSE54460 dataset (Figure 5G). Moreover, the ROC curve showed good performance of these models for survival prediction in the TCGA dataset, and the area under the curve (AUC) was 0.725 at 1 year, 0.705 at 2 years, 0.743 at 3 years, and 0.745 at 5 years (Figure 5B). The efficiency of the risk model was further validated in the GSE21034 (Figure 5E), GSE54460 (Figure 5H), GSE70768 (Figure 5K), and GSE70769 (Figure 5N) datasets and showed good performance. PCA was performed to determine the distribution characteristics of the high-risk and low-risk groups. Different distributions for high-risk and low-risk patients were confirmed in the TCGA (Figure 5C), GSE21034 (Figure 5F), GSE54460 (Figure 5I), GSE70768 (Figure 5L), and GSE70769 (Figure 5O) datasets.

### Associations Between the Prognostic Signature and Clinicopathological Factors of PCa

**FIGURE 6:**
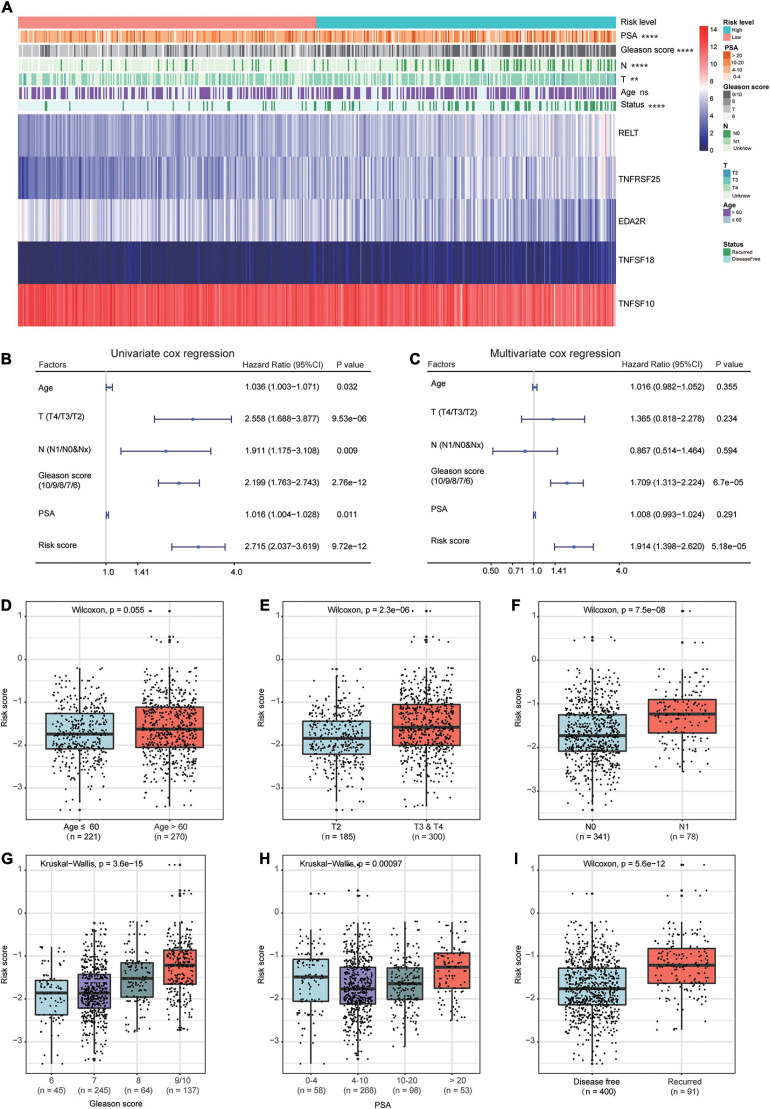
Relationship between the prognostic signature and clinicopathological factors of PCa patients. **(A)** The heat map shows the expressions of the five costimulatory molecule genes and clinicopathological factors in the high- and low-risk groups. The univariate **(B)** and multivariate **(C)** Cox regression analyses of clinicopathological factors (including the risk score) and prognosis. The bar chat showed that the prognostic signature had significantly different in different clinical subgroups, and the PCa patients with advanced age **(D)**, high pathological T stage **(E)**, node metastasis **(F)**, high Gleason score **(G)**, high prostate-specific antigen **(H)**, and recurrence **(I)** tend to have a high risk score. ns: *P* > 0.05, *P < 0.05, **P < 0.01, ***P < 0.001, ****P < 0.0001.

The heat map shows the expression of the five selected survival-related costimulatory molecule genes and clinicopathological factors in the high- and low-risk groups (Figure 6A). Moreover, the detailed distribution of the clinicopathological data across patient subgroups is shown in Table 2. The results showed that high-risk patients tended to have an advanced T stage, high prostate-specific antigen (PSA) levels, high Gleason scores and lymphatic metastasis. We used univariate and multivariate Cox regression analyses to determine whether the prognostic signature was an independent predictor factor for disease free survival in PCa patients. Univariate Cox regression analyses showed that patient age, pathological T stage, pathological N stage, Gleason score, PSA level, and risk score were significantly associated with prognosis (Figure 6B). Multivariate Cox regression analyses revealed that the Gleason score and risk score were significantly associated with prognosis (Figure 6C). These results demonstrated that the prognostic signature is an independent risk factor that can predict the prognosis of PCa patients. To determine the relationship between the risk signature and clinicopathological factors (age, pathological T stage, pathological N stage, Gleason score, and PSA level), patients were separated into different subgroups according to clinicopathological variables. The bar charts show that PCa patients with advanced age (Figure 6D), high pathological T stage (Figure 6E), lymph node metastasis (Figure 6F), high Gleason score (Figure 6G), high PSA levels (Figure 6H), and recurrence (Figure 6I) tended to have a high risk score. These results demonstrated that our prognostic signature is closely associated with the clinical factors of PCa.

**TABLE 2:** Association between the risk score model and patients’ clinical characteristics.

Variables	TCGA cohort (*n* = 491), n (%)	Risk score		*P*-value
		Low risk (*n* = 233)	High risk (*n* = 258)	
Age (mean ± SD, years)	61.0 ± 6.8	60.3 ± 6.9	61.6 ± 6.7	0.045
≤60 years	221 (45.0)	115 (49.4)	106 (41.1)	0.070
>60 years	270 (55.0)	118 (50.6)	152 (58.9)	
**Pathological T stage**				0.001
T2	185 (37.7)	108 (46.4)	77 (29.8)	
T3	290 (59.1)	120 (51.5)	170 (65.9)	
T4	10 (2.0)	3 (1.3)	7 (2.7)	
Unknown	6 (1.2)	2 (0.8)	4 (1.6)	
Nodal stage				<0.001
N0	341 (69.4)	173 (74.2)	168 (65.1)	
N1	78 (15.9)	19 (8.2)	59 (22.9)	
Unknown	72 (14.7)	41 (17.6)	31 (12.0)	
**Gleason score**				<0.001
6	45 (9.2)	28 (12.1)	17 (6.6)	
7	245 (49.9)	150 (64.4)	95 (36.8)	
8	64 (13.0)	22 (9.4)	42 (16.3)	
9	134 (27.3)	33 (14.1)	101 (39.1)	
10	3 (0.6)	0 (0)	3 (1.2)	
**PSA at initial diagnosis (ng/ml)**				0.011
≤4	58 (11.8)	22 (9.4)	36 (14.0)	
(4,10]	268 (54.6)	144 (61.8)	124 (48.1)	
(10,20)	98 (19.9)	45 (19.3)	53 (20.5)	
>20	53 (10.8)	16 (6.9)	37 (14.3)	
Unknown	14 (2.9)	6 (2.6)	8 (3.1)	

### Prognostic Signature-Related Biological Processes and Pathways

**FIGURE 7:**
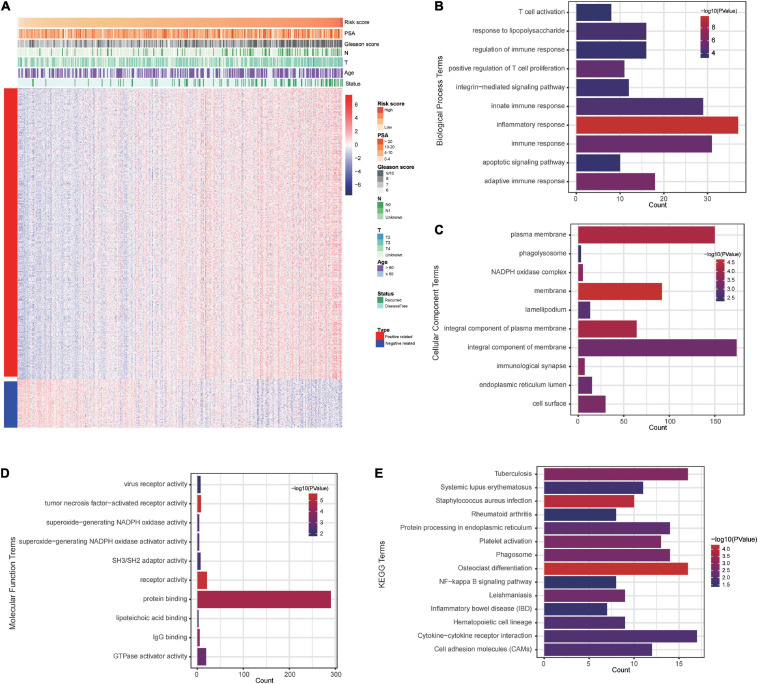
The costimulatory molecule-based signature-related biological pathways. **(A)** The most related genes of costimulatory molecule-based signature in PCa (Pearson | *R*| > 0.4). **(B–E)** The GO and KEGG analysis of the identified the potential functions and pathways of costimulatory molecule genes.

To explore signature-related biological pathways, genes strongly correlated with the risk score were filtered. A total of 525 positively related genes and 87 negatively related genes in the TCGA dataset (Pearson | *R*| > 0.4) were obtained (Figure 7A). The detailed results of the functional enrichment analysis are shown in Supplementary Table 2. The top ten results of the GO analyses are shown in Figures 7B–D. The most enriched terms for biological process, cellular components, and molecular function were “inflammatory response,” “membrane,” and “tumor necrosis factor-activated receptor activity,” respectively. According to the KEGG analysis, the most significantly enriched term was osteoclast differentiation (Figure 7E).

### The Prognostic Signature Was Associated With the Immune Microenvironment

**FIGURE 8:**
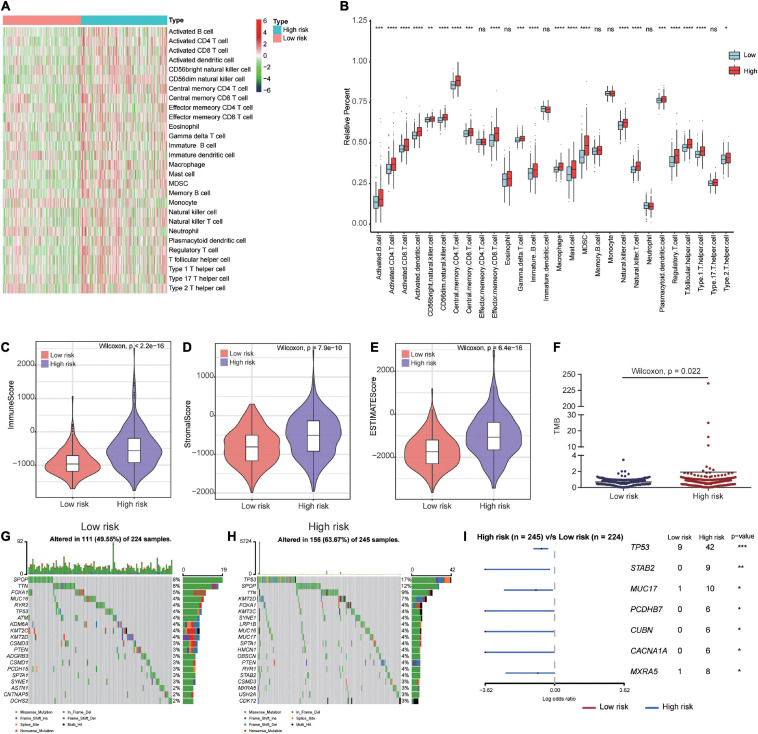
Immune microenvironment and tumor mutation burden in high-risk and low-risk patients. **(A)** The gene expression profile of 28 immune cell types in high- and low-risk patients. **(B)** Box plots showing 28 differential immune cell infiltration difference between high- and low-risk patients. **(C–E)** The immune score, stromal score and ESTIMATE score in high- and low-risk patients. **(F)** Patients in high-risk group had higher tumor mutation burden than those in low-risk group. **(G,H)** The mutation profile of the top 20 mutation genes in the low- and high-risk groups. **(I)** Forest plot illustrated the differences of mutation frequency of several gene (TP53, STAB2, MUC17, PCDHB7, CUBN, CACNA1A, and MXRA5) in high- and low-risk patients. *P < 0.05, **P < 0.01, ***P < 0.001, ****P < 0.0001.

To gain a better understanding of the associations between our prognostic signature and the immune microenvironment, we displayed the expression profiles of 28 immune cell types in a heat map for high- and low-risk groups. The results showed significant differences in the immune cell infiltration status between high- and low-risk patients (Figure 8A). The box plots showed that high-risk patients had a high percentage of immune cells, including activated B cells, activated CD8 T cells, activated CD4 T cells, CD56bright natural killer cells, CD56dim natural killer cells, central memory CD4 T cells, central memory CD8 T cells, immature B cells, natural killer cells, type 1 T helper cells, activated dendritic cells, gamma delta T cells, macrophages, mast cells, myeloid-derived suppressor cells (MDSCs), plasmacytoid dendritic cells, regulatory T cells, T follicular helper cells, and type 2 T helper cells (Figure 8B). The immune and stromal scores for the TCGA cohort were calculated according to the ESTIMATE algorithm. We found that high-risk PCa patients had significantly higher immune scores, stromal scores, and ESTIMATE scores than low-risk patients (Figures 8C–E).

### Differences in Genomic Alterations Between High-Risk and Low-Risk Patients

To investigate whether there were differences in genomic alterations between the 2 clusters, we compared the TMB and gene mutation patterns between high-risk and low-risk patients with available somatic mutation data in the TCGA entire set. Patients in the high-risk group had a higher TMB than those in the low-risk group (Figure 8F). The top 20 mutated genes for low-risk (Figure 8G) and high-risk (Figure 8H) patients were compared. We found that 49.55% of samples in the low-risk group and 63.67% of samples in the high-risk group had alterations in these genes. The forest plot illustrated that the TP53, STAB2, MUC17, PCDHB7, CUBN, CACNA1A, and MXRA5 genes were mutated at a significantly higher rate in high-risk patients (Figure 8I).

## Discussion

Immunotherapy, as a rapidly growing field, aims to identify corresponding biomarkers prompting the immune system to recognize and kill cancer cells (Bilusic et al., 2017; Cha et al., 2020). Accumulating evidence indicated that the potential of immunotherapy in PCa treatment (Bilusic et al., 2017). However, effective biomarkers for predicting the prognosis of PCa patients remain scarce, and a single biomarker is insufficient to predict the clinical outcomes and response to immunotherapy (Smits et al., 2017). Previous studies have revealed that costimulatory molecules play an important role in the progression of various tumors (Loos et al., 2008; Zou and Chen, 2008; Geng et al., 2015). To improve the clinical therapy outcomes of PCa, we identified five costimulatory molecules and constructed a new prognostic signature for PCa patients based on these five genes. To our knowledge, our study provides the first prognostic signature of costimulatory molecules in patients with PCa. Moreover, the efficiency of our signature was well validated in four different GEO datasets. The multivariate Cox regression analysis indicated that our signature was an independent predictive factor for PCa patients and was significantly correlated with prognosis in different clinical subgroups. These results demonstrated that our prognostic signature is an effective and reliable prognostic tool. Additionally, we found that our prognostic signature was associated with the immune microenvironment. These findings should enhance the development of immunotherapeutic strategies for PCa patients.

The costimulatory molecules play an important role in the regulation of tumor immunity (Pitt et al., 2016). Topalian et al. (2020) reported that the costimulatory molecules expressed on tumor cells or lymphocytes play vital roles in regulating the antitumor immune response and that these molecules are closely related to the progression of tumors. At present, there are two main families of costimulatory molecules: the B7-CD28 family and the TNF family (Tang et al., 2018; Dostert et al., 2019). To determine the expression of the costimulatory molecule genes in PCa patients, we acquired data on 13 B7-CD28 family members and 47 TNF family members for our study (Janakiram et al., 2015; Dostert et al., 2019). After using univariate and multivariate Cox proportional hazards regression analyses, we identified five costimulatory molecular genes (RELT, TNFRSF25, EDA2R, TNFSF18, and TNFSF10) with prognostic value. However, the functions and roles of these costimulatory molecules in PCa remain unclear. RELT, a new member of the TNFRSF family, accelerates tumor progression and regulates the infiltration of numerous immune cell types. RELT is significantly upregulated as glioma grade increases and is associated with a poor prognosis (Jin et al., 2020). TNFRSF25, also known as death receptor 3 (DR3), belongs to the TNFRSF family and is highly expressed in T cells. Recent studies have shown that DR3 is a potential immunotherapy molecular target for cancer treatment and plays essential roles in protective inflammation, autoimmune diseases, and tumor immunotherapy (Mavers et al., 2019; So and Ishii, 2019). EDA2R, also known as TNFRSF27 or XEDAR, mainly coordinates various cellular and organismal biological processes and exerts its roles by activating gene transcription. Studies have indicated that EDA2R is a direct p53 target that can be activated by p53 (Brosh et al., 2010). They also confirmed that treatment of cancer cells with the ligand EDA-A2, which can specifically activate EDA2R, leads to p53-dependent cell death. In addition, Tanikawa et al. (2010) found that EDA2R is frequently downregulated in colorectal cancer patients due to epigenetic alterations. Interestingly, this is consistent with our findings that EDA2R is also markedly downregulated in PCa patients in the TCGA dataset. TNFSF18 is one of the TNFRSF members expressed by myeloid cells and provides costimulatory signals to boost T cell activity. The blockade of TNFSF18-GITR signaling to target mesothelioma stem cells might be translated into a therapeutic strategy for mesothelioma treatment (Wu et al., 2019). Recent evidence has further shown that TNFSF10 is an apoptosis-inducing ligand and can promote the apoptosis of cancer cells (He et al., 2012). Sun et al. (2018) revealed that the abnormal expression of TNFSF10 influences the apoptosis of PCa cells. Furthermore, relevant studies have shown that TNFSF10 is a promising anticancer agent for cancer that exhibits good anticancer activity by inducing apoptosis (Janakiram et al., 2015; Dostert et al., 2019).

Based on these five costimulatory molecular genes, we developed a prognostic signature for PCa patients. The performance of our prognostic signature was validated in four different independent GEO datasets. The results showed good performance in three datasets (GSE21034, GSE70768, and GSE70769). The *P-*value in the GSE54460 dataset did not reach statistical significance, which might be attributed to the small sample size. In addition, we further investigated the association between the prognostic signature and clinical factors. We found the high-risk patients were older and had more advanced T stage, a higher rate of lymphatic metastasis, higher Gleason scores, higher PSA levels and a higher mortality rate than low-risk patients. These results revealed that our prognostic signature is closely correlated with clinical factors. Therefore, this prognostic signature could be applied as a supplement for guiding treatment and improving the clinical prognosis of PCa patients. In general, our prognostic signature was well validated in different GEO datasets, which encouraged our investigation of the underlying molecular mechanisms. Through GO and KEGG enrichment analysis of these costimulatory molecules, we found that the potential molecular mechanisms of these costimulatory molecules in PCa were closely related to the immune pathway, as indicated by the enrichment of terms such as “regulation of immune response,” “innate immune response,” “immune response,” “adaptive immune response,” “immunological synapse,” and “positive regulation of T cell proliferation.” These results indicate that immune heterogeneity may be the cause of the difference in prognosis between patient groups.

Subsequently, we explored the immune cell infiltration and tumor mutation profiles of the high-risk and low-risk groups to further reveal the difference in the immune microenvironment between these two groups. The results showed that the high-risk group exhibited significantly greater infiltration of immune cells, including activated B cells, activated CD8 T cells, activated CD4 T cells, CD56bright natural killer cells, CD56dim natural killer cells, central memory CD4 T cells, central memory CD8 T cells, immature B cells, natural killer cells and type 1 T helper cells, than the low-risk group. In addition, the infiltration of various immunosuppressive cells, including activated dendritic cells, gamma delta T cells, macrophages, mast cells, MDSCs, plasmacytoid dendritic cells, regulatory T cells, T follicular helper cells, and type 2 T helper cells, was greater in high-risk patients than in low-risk patients, suggesting the presence of an immunosuppressive microenvironment in high-risk patients. Complex interactions between immunosuppressive cells cooperate to suppress antitumor immune responses and promote disease progression. Targeting regulatory T cell function or the secretion of immunological processes can lead to tumor immune evasion (Vinay et al., 2015). Su et al. (2019) found that CCL2 induced the recruitment of M2-like tumor-associated macrophages and regulatory T cells, thus promoting metastasis with immune suppression and neoangiogenesis in PCa. Sullivan et al. (2021) studied Hi-Myc mice crossed to mast cell knockout mice and demonstrated that higher levels of mast cell infiltration led to the promotion of cancer cell invasion. MDSCs are known to play critical roles in tumor immune evasion. Lu et al. (2017) found that MDSCs promoted the initiation and progression of PCa in a mouse model. In addition, the level of MDSC infiltration correlated with the PSA levels and metastasis in PCa patients (Lu et al., 2017). Understanding the immune microenvironment of each PCa patient can help us identify patients who are more likely to benefit from immunotherapy.

Similarly, we found that the tumor mutation burden (TMB) in the high-risk group was also higher than that in the low-risk group, especially in terms of TP53 mutation frequency, which was obviously increased in the high-risk group. Some scholars have found that sequencing technology indicates that the TP53 mutation frequency is much higher than that reported in the TCGA database (Mateo et al., 2020). Martin et al. (2011) revealed that prostate epithelial Pten/TP53 loss leads to epithelial to mesenchymal transition. TP53 mutation is linked to a higher level of tumor-infiltrating T cells, which influences the immunotherapy response in prostate cancer (Kaur et al., 2019). Mu et al. (2017) reported that PTEN loss with TP53 mutation causes resistance to antiandrogen therapy of PCa. In addition, Jiang et al. (2018) demonstrated that TP53 mutation could result in an immunosuppressed state in gastric cancer. In the present study, the immunosuppressive microenvironment in high-risk patients might be partly due to the high frequency of TP53 mutations in high-risk patients. The above conclusions revealed that these costimulatory molecules have important prognostic values for patients with PCa. These findings give us sufficient confidence that our signature can be applied as a novel strategy for guiding treatment and improving clinical therapy outcomes.

In our study, we constructed a costimulatory molecule-related prognostic signature for PCa, which could be used to stratify patients to further guide treatment and improve clinical outcomes. This study was the first comprehensive study of the expression profiles and clinical significance of costimulatory molecule genes in PCa patients. Although our study provides important insights to better evaluate costimulatory molecules and the prognosis of PCa patients, it inevitably has some limitations that need to be noted. First, regardless of the fact that we used four different independent datasets for validation, the present study was a retrospective study. All data were obtained from the public databases. Moreover, our research was entirely conducted through a series of bioinformatics methods, so experimental and prospective studies are needed to further confirm the good predictive ability of our prognostic signature.

## Conclusion

In this study, we performed the first comprehensive analysis of costimulatory molecules in patients with PCa through a series of bioinformatics analysis methods. We identified several key prognostic costimulatory molecule genes, built a reliable and valid prognostic signature, and explored the potential molecular mechanisms of this signature. Our prognostic signature could stratify PCa patients into two subgroups with different prognoses and showed high associations with the clinical features. Moreover, patients identified as high risk based on our prognostic signature exhibited a high mutation frequency, a high level of immune cell infiltration, and an immunosuppressive microenvironment. Thus, our signature could provide clinicians with prognosis predictions and treatment guidance for PCa patients.

## Data Availability Statement

The datasets presented in this study can be found in online repositories. The names of the repository/repositoriesand accession number(s) can be found in the article/Supplementary Material.

## Author Contributions

CL and ST conceived and designed the experiments. SG, XH, and JC acquired and analyzed the data. SG and XH wrote this manuscript. HX checked the analysis procedure. LZ and JZ checked the manuscript. All the authors read and approved the final manuscript.

## Conflict of Interest

The authors declare that the research was conducted in the absence of any commercial or financial relationships that could be construed as a potential conflict of interest.

## Publisher’s Note

All claims expressed in this article are solely those of the authors and do not necessarily represent those of their affiliated organizations, or those of the publisher, the editors and the reviewers. Any product that may be evaluated in this article, or claim that may be made by its manufacturer, is not guaranteed or endorsed by the publisher.

## Footnotes

1
https://xenabrowser.net/

2
http://cbioportal.org

3
https://www.ncbi.nlm.nih.gov/geo/

4
https://david.ncifcrf.gov/

5
https://portal.gdc.cancer.gov/repository

## Supplementary Material

Supplementary Figure 1 The relationship between the costimulatory molecules. Red represents a negative correlation, and blue represents a positive correlation.

Supplementary Figure 2 Selection of most valuable costimulatory molecule genes for prostate cancer. **(A,B)** The coefficients of LASSO Cox regression analysis to identify the most valuable prognostic genes were showed.

Supplementary Figure 3 Identification of cluster numbers using consensus clustering. **(A)** Consensus clustering matrix for *k* = 2. **(C)** Consensus clustering matrix for *k* = 3. **(E)** Consensus clustering matrix for *k* = 4. **(G)** Consensus clustering matrix for *k* = 5. Principal component analysis for evaluating the distributions of different cluster numbers: **(B)** two clusters; **(D)** three clusters; **(F)** four clusters; **(H)** five clusters.

## Abbreviations

PCa: prostate cancer
mCRPC: metastatic castrate resistant prostate cancer
ICIs: immune checkpoint inhibitors
TME: tumor microenvironment
TNF: tumor necrosis factor
PD-1: programmed cell death protein 1
PD-L1: programmed cell death 1 ligand 1
TNFSF: tumor necrosis factor ligands superfamily
TNFRSF: tumor necrosis factor receptor superfamily
TCGA: The Cancer Genome Atlas
GEO: Gene Expression Omnibus
RMA: robust multi-array average
RSEM: RNA-Seq by Expectation-Maximization
LASSO: least absolute shrinkage and selection operator
GSEA: Gene Set Enrichment Analysis
FDR: false discovery rate
PCA: principal component analysis
ROC: receiver operating characteristic
GO: gene ontology
KEGG: Kyoto Encyclopedia of Genes and Genomes
ssGSEA: single-sample gene set enrichment analysis
TMB: tumor mutation burden
AUC: area under the curve
PSA: prostate-specific antigen
DR3: death receptor 3
MDSCs: myeloid-derived suppressor cells.
