# Supplementary figures and images for: Identification of a Costimulatory Molecule-Related Signature for Predicting Prognostic Risk in Prostate Cancer
Source: Front Genet. 2021 Aug 16;12:666300. doi: 10.3389/fgene.2021.666300 (PMC8415313; doi:10.3389/fgene.2021.666300)

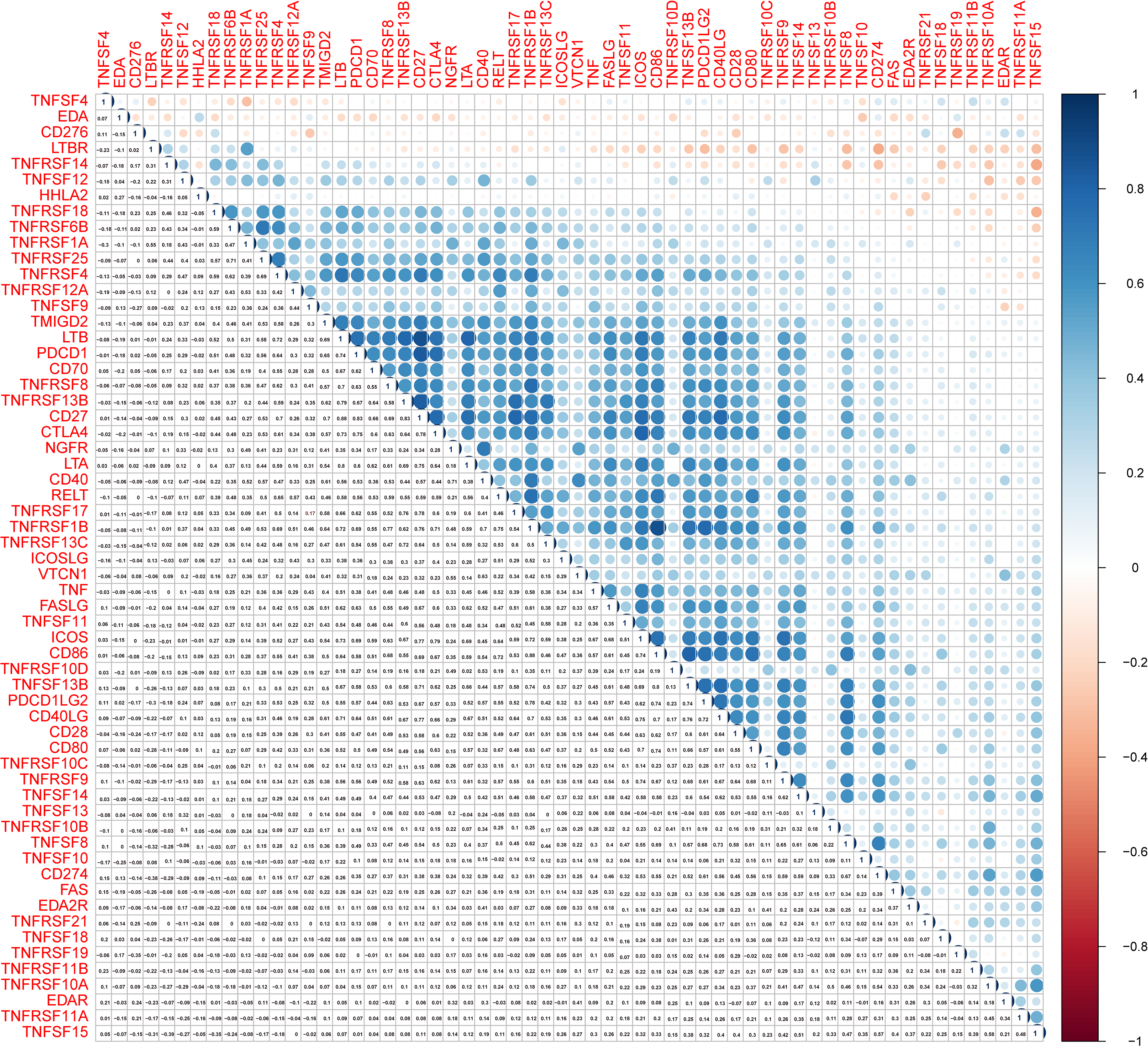

Supplement: Supplementary Figure 1 — The relationship between the costimulatory molecules. Red represents a negative correlation, and blue represents a positive correlation. [file Image_1.TIF]

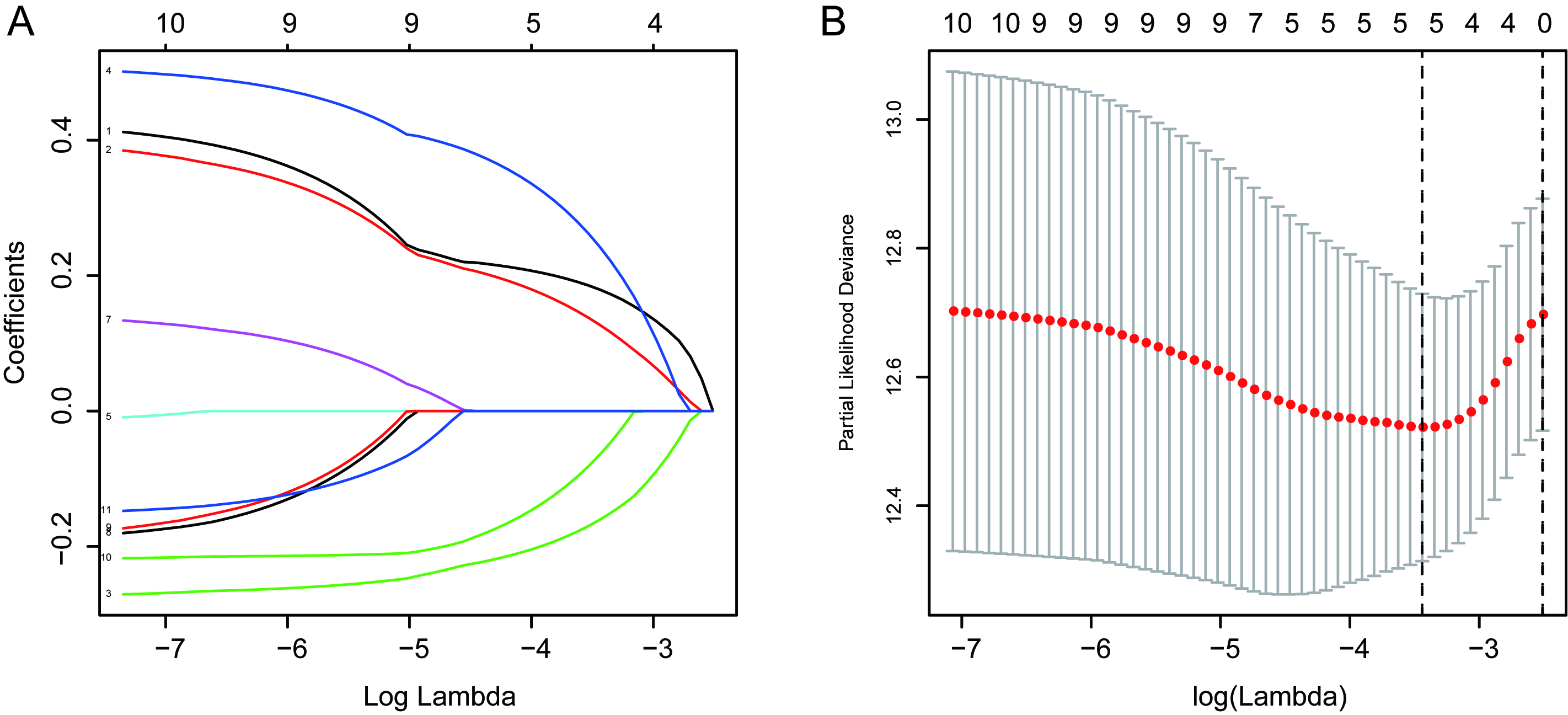

Supplement: Supplementary Figure 2 — Selection of most valuable costimulatory molecule genes for prostate cancer. (A,B) The coefficients of LASSO Cox regression analysis to identify the most valuable prognostic genes were showed. [file Image_2.TIF]

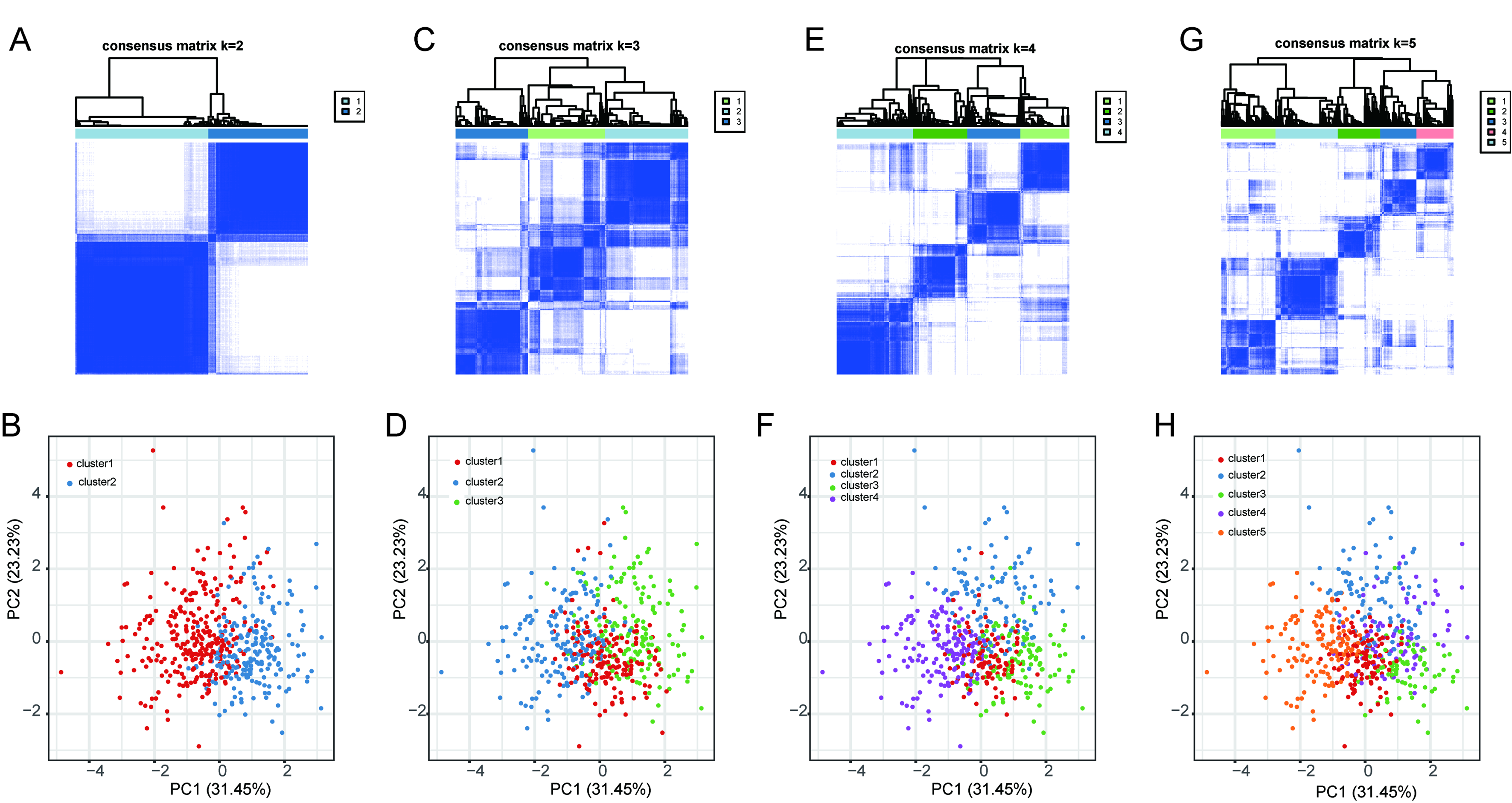

Supplement: Supplementary Figure 3 — Identification of cluster numbers using consensus clustering. (A) Consensus clustering matrix for k = 2. (C) Consensus clustering matrix for k = 3. (E) Consensus clustering matrix for k = 4. (G) Consensus clustering matrix for k = 5. Principal component analysis for evaluating the distributions of different cluster numbers: (B) two clusters; (D) three clusters; (F) four clusters; (H) five clusters. [file Image_3.TIF]
