# Supplementary material for: Identification of a Costimulatory Molecule-Related Signature for Predicting Prognostic Risk in Prostate Cancer
Source: Front Genet. 2021 Aug 16;12:666300. doi: 10.3389/fgene.2021.666300 (PMC8415313; doi:10.3389/fgene.2021.666300)
Supplement: Supplementary file 4 [file Table_1.DOCX]

**Table S1. Publicly-available datasets used in the study.**

| **Dataset** | **Samples** | **Data Array** | **Sample Description** | **Reference** |
| --- | --- | --- | --- | --- |
| TCGA | 550 | RNA-seq | 52 normal samples and 498 PCa samples | <https://xenabrowser.net/> |
| GSE21034 | 140 | Affymetrix Human Exon 1.0 ST Array | 140 PCa samples | Taylor BS et al. Cancer Cell. 2010;18(1):11-22 |
| GSE54460 | 90 | Illumina HiSeq 2000 | 90 PCa samples | Long Q et al. Cancer Res. 2014;74(12):3228-37 |
| GSE70768 | 111 | Illumina HumanHT-12 V4.0 expression beadchip | 111 PCa samples | Ross-Adams H et al. EBioMedicine. 2015;2(9):1133-44 |
| GSE70769 | 92 | Illumina HumanHT-12 V4.0 expression beadchip | 92 PCa samples | Ross-Adams H et al. EBioMedicine. 2015;2(9):1133-44 |

Abbreviations: GSE, data set accession ID in Gene Expression Omnibus; TCGA, The Cancer Genome Atlas; PCa, prostate cancer.
